# Circulating tumor nucleic acids: biology, release mechanisms, and clinical relevance

**DOI:** 10.1186/s12943-022-01710-w

**Published:** 2023-01-21

**Authors:** Pavel Stejskal, Hani Goodarzi, Josef Srovnal, Marián Hajdúch, Laura J. van ’t Veer, Mark Jesus M. Magbanua

**Affiliations:** 1 Institute of Molecular and Translational Medicine, Faculty of Medicine and Dentistry, Palacký University and University Hospital in Olomouc, Olomouc, 779 00 Czech Republic; 2 Department of Biochemistry and Biophysics, University of California San Francisco, San Francisco, CA 94158 USA; 3 Department of Urology, University of California San Francisco, San Francisco, CA 94158 USA; 4 Department of Laboratory Medicine, University of California San Francisco, 2340 Sutter Street, San Francisco, CA USA

**Keywords:** Circulating tumor DNA, Circulating tumor RNA, Cell-free DNA, Shedding mechanisms, Liquid biopsy, Biomarkers, Precision oncology, Clinical application

## Abstract

**Background:**

Despite advances in early detection and therapies, cancer is still one of the most common causes of death worldwide. Since each tumor is unique, there is a need to implement personalized care and develop robust tools for monitoring treatment response to assess drug efficacy and prevent disease relapse.

**Main body:**

Recent developments in liquid biopsies have enabled real-time noninvasive monitoring of tumor burden through the detection of molecules shed by tumors in the blood. These molecules include circulating tumor nucleic acids (ctNAs), comprising cell-free DNA or RNA molecules passively and/or actively released from tumor cells. Often highlighted for their diagnostic, predictive, and prognostic potential, these biomarkers possess valuable information about tumor characteristics and evolution. While circulating tumor DNA (ctDNA) has been in the spotlight for the last decade, less is known about circulating tumor RNA (ctRNA). There are unanswered questions about why some tumors shed high amounts of ctNAs while others have undetectable levels. Also, there are gaps in our understanding of associations between tumor evolution and ctNA characteristics and shedding kinetics. In this review, we summarize current knowledge about ctNA biology and release mechanisms and put this information into the context of tumor evolution and clinical utility.

**Conclusions:**

A deeper understanding of the biology of ctDNA and ctRNA may inform the use of liquid biopsies in personalized medicine to improve cancer patient outcomes.

## Background

Despite advances in early detection and treatment, the number of new cancer cases and deaths is still increasing globally [1]. Moreover, each tumor possesses a unique genetic profile and has the potential to develop drug resistance and spread to distant sites [2]. Hence, new strategies for personalized treatment guided by diagnostic, predictive, and prognostic biomarkers are urgently needed to reverse increasing incidence and mortality rates. Adopting blood-based liquid biopsy into clinical practice could help guide therapeutic strategies in personalized medicine. Robust and accessible biomarkers for immediate assessment of tumor response and monitoring of minimal residual disease (MRD) are crucial to improving patient outcomes. Thus, recently published research articles and reviews have highlighted the potential of liquid biopsy-based biomarkers as a real-time reflection of the tumor burden with diagnostic, prognostic, and predictive information to guide cancer management [2–8].

Cells and DNA shed by tumors into circulation, also known as circulating tumor cells (CTCs) and circulating tumor DNA (ctDNA), respectively, are considered two major components of liquid biopsy [9]. However, the lack of standardization of CTC detection methods [10], as well as the high false-negative rate of ctDNA assays, points to the need for further technological advancements to support liquid biopsy standardization and improve test performance [9]. Circulating tumor RNA (ctRNA) is an emerging biomarker that could provide unique information not found in CTCs and ctDNA.

Cell-free DNA (cfDNA) are fragments of DNA released into the bloodstream which originate mainly from the apoptosis of hematopoietic cells [6, 8]. DNA released by tumor cells may possess alterations that can provide highly specific markers for detection [6, 8, 11]. Notably, compared to healthy individuals, cancer patients’ blood has been observed to contain increased levels of cfDNA [12] as well as messenger RNA (mRNA) and non-coding RNA (ncRNA) [13, 14]. Cell-free nucleic acids (cfNAs) can be released passively into circulation mainly via apoptosis and necrosis as well as through active secretion via extracellular vesicles (EVs) from viable cells. In this review, we use the term ctNAs to represent the fraction of total cfNAs (DNA and RNA) released exclusively by tumor cells. Understanding the nature and origin of ctNAs provides pivotal clues for exploiting these biomarkers in specific clinical settings. The unique characteristics of ctNA molecules go hand in hand with the process of their release from cells and the kinetics of their clearance [15, 16]. While ctDNA can harbor critical genetic traits of tumorigenesis, ctRNA can reflect intra-tumoral dynamic processes on the cellular and intercellular levels [4, 17].

Aside from blood, other non-invasive approaches using urine, saliva, and semen plasma, along with invasive methods using cerebrospinal fluid (CSF), and pleural and peritoneal effusions, have been utilized to assess ctNAs [18–20]. A recent review article discussed the properties of ctDNAs originating from different body fluids providing a comprehensive summary of the peculiarities of ctDNA from various sources [20]. For example, ctDNAs detected in urine are composed of shorter fragments (< 100 bp) that are passed from plasma through the glomeruli (tiny networks of blood vessels in the kidney involved in waste filtration) as well as longer ctDNA fragments shed directly by tumor cells in the urinary tract [20, 21]. Additionally, saliva has been suggested as a potential source of ctDNA from local tumors but is very short (40–60 bp) and less enriched [20]. The concentration of ctDNA can also vary from one compartment to another. For example, ctDNA concentration in the CSF is higher than in the plasma [22], perhaps due to the presence of fewer immune cells (compared to blood) that could contribute to the background cfDNA. Similarly, pleural and peritoneal effusions comprise a richer source of ctDNA than plasma due to the proximity of these fluids to tumors that shed these molecules [20, 23]. In addition, cfDNA levels are relatively higher in seminal plasma than other body fluids depending on sexual activity/abstinence and individual composition of the seminal fluid [20, 24].

Despite the numerous publications on ctNAs, there are still many unanswered questions. What governs the fluctuations in the ctNA levels in the blood? Is it possible to distinguish between ctNAs shed by cells dying in response to treatment and ctNAs actively secreted by treatment-resistant cells? Is the absence of ctNAs in cancer patients due to the assay’s low sensitivity (false negativity), or can these biomarkers be truly absent, and why? And, if detected, why do they not correlate, in some cases, with tumor pathophysiologies such as size and stage? And how big an issue is false positivity? To adopt ctNA assays in clinical practice, we need to understand not only their nature and mechanisms of release from cells but also their fates in circulation.

Many research articles and reviews have focused on cfDNA or ctDNA, but less is known about ctRNA. This review summarizes the biology of ctDNA and ctRNA, their release mechanisms from cells, and the kinetics of degradation. Finally, we put these findings in the context of cancer evolution and clinical utility.

## Circulating tumor nucleic acid release mechanisms

**Fig. 1:**
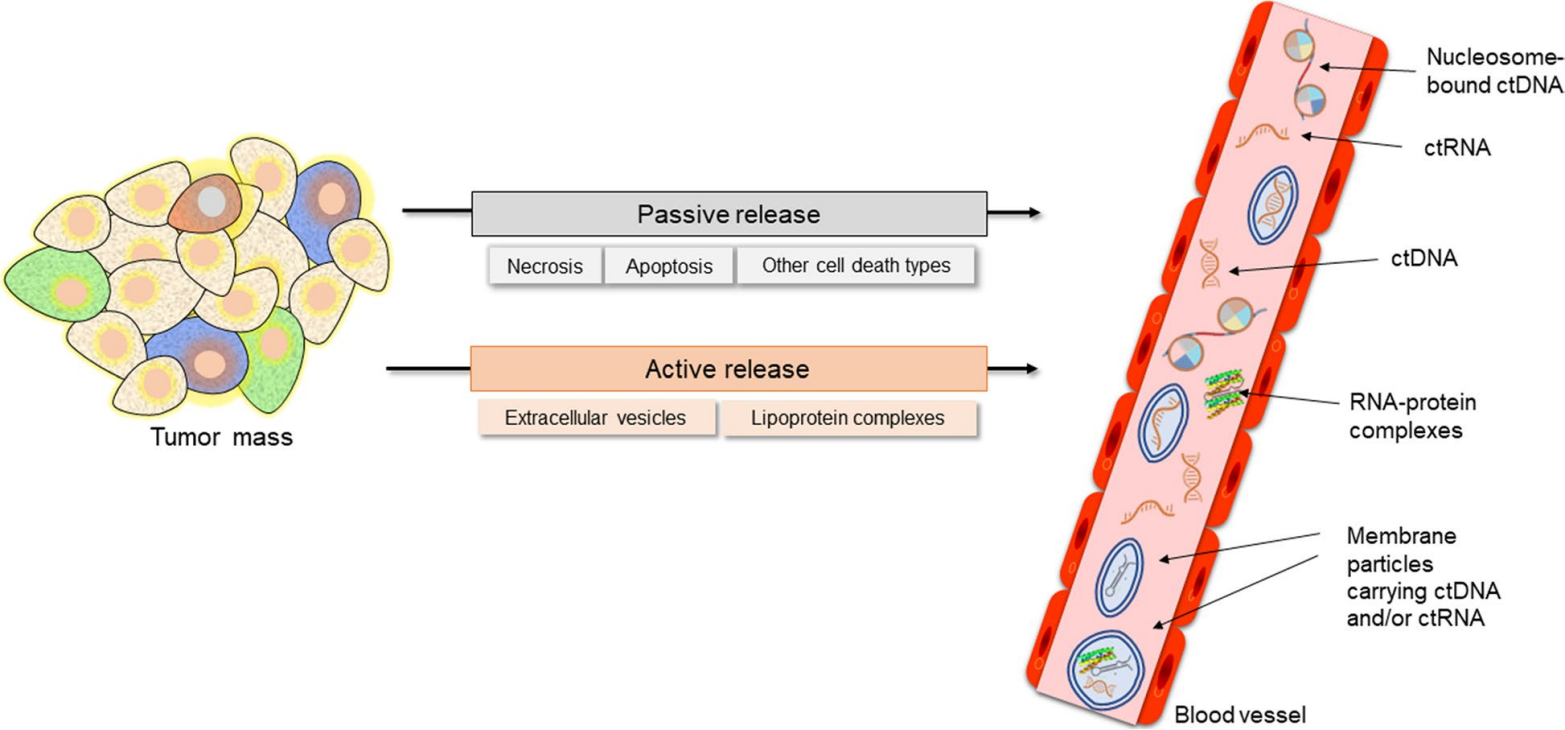
Circulating tumor nucleic acid release mechanisms. Circulating tumor nucleic acids may be released passively from tumor bed cells as free or protein-associated fragments or actively as part of extracellular vesicles and lipoprotein complexes

Mechanisms involved in ctNA release need to be better understood. There are considerable gaps in our knowledge regarding the presence, fluctuations, and characteristics of ctNAs and their potential roles in tumor resistance and evolution. Recent improvements in the sensitivity and specificity of detection methods [10, 25] have facilitated the progress in understanding the biology of ctDNA [6] and ctRNA [14]. While these data could be divergent, burdened with preanalytical variabilities, and lacking standardization methods [26], unified themes can be gleaned from the information available (Fig. 1). To date, systematic investigations of active and passive ctNA release mechanisms have yet to be, to our knowledge, fully described. In the following section, we discuss the current findings about the mechanisms involved in the release of ctNAs into circulation.

### Passive release mechanisms of circulating tumor nucleic acids and their properties

Hematopoietic cell turnover has been suggested as the most significant source of cfDNA in blood and is related mainly to cell death by apoptosis [6, 8]. Cancer cells can undergo cell death, either by apoptosis or necrosis, resulting in the release of ctDNA [15]. Apoptosis and necrosis are considered major contributors to ctDNA release, but their exact contribution is unknown [6, 11, 27]. Uncontrolled proliferation is a well-known characteristic of cancer. Rapid cell proliferation causes local nutrient depletion, hypoxia, inflammation, oxidative stress, acidosis, and the production of corresponding tissue-specific transcription factors and signaling death-inducing molecules [28, 29]. Apoptosis and necrosis are major results of hypoxic and metabolic stress and can cause the passive release of cellular content into the extracellular space [11, 30]. In this section, we discuss processes involved in the passive release of ctDNA and summarize current knowledge about passive ctRNA release, a much less understood phenomenon than ctDNA release.

#### Apoptotic cell-derived cell-free DNA possesses a ladder-like pattern profile

Apoptosis is a form of programmed cell death for maintaining homeostatic balance and is executed by caspases, leading to cell shrinkage, chromatin condensation, and fragmentation. Cellular contents, including nucleic acids (NAs), are then packaged into apoptotic bodies protecting them from digestion by circulating nucleases. The apoptotic bodies are then cleared by phagocytosis, enzymatically digested, and released as soluble debris [4, 31].

Although the exact proportions of NAs released via different types of cell death are unknown, some characteristics can indicate the source of cfNAs. There is strong evidence that cfDNA fragment size distribution is not random and possesses a ladder-like pattern of sizes as visualized by gel electrophoresis [32]. CfDNA fragment size depends on the number of nucleosomes the DNA is wrapped around [5, 11]. The peak cfDNA fragment size is 167 bp, corresponding to the length of DNA around one nucleosome (147 bp) and a linker DNA (20 bp) protecting DNA from cleavage [33–35]. This characteristic of cfDNA is predominantly a result of apoptotic internucleosomal DNA fragmentation. Briefly, caspase-activated DNase (CAD) [36], DNaseI L-3 [37], NM23-H1 [38], and EndoG [39] nucleases are activated after apoptotic stimuli and execute continual DNA fragmentation with specificity for the internucleosomal region of DNA not protected by histones. A subset of cfDNA can still be wrapped around histones and thus circulate as nucleosomes [33]. CfDNA wrapped around nucleosomes are protected from cleavage by DNases [33–35].

#### Necrotic tumor cells release DNA mainly through phagocytosis

Besides apoptosis, necrosis is also considered a significant source of cfDNA in cancer patients [5, 15]. Unlike apoptosis, the purpose of which is to eliminate abnormal or unneeded cells, necrosis is a faster and more direct reflection of the adverse tumor environment in cancer. Necrotic cells exhibit organelle dysfunction and plasma membrane aberration, which may lead to the random release of cellular components exposing the tumor DNA to intracellular and extracellular degradative agents such as nucleases and free radicals [4, 5, 27]. As a result of the non-systematic release and digestion of DNA during necrosis, larger fragments of up to many kilo-base pair (kbp) sizes are thought to be shed in circulation [4, 11]. The resulting sizes are useful characteristics for identifying necrosis-derived cfDNA [4].

Necrosis-induced release of DNA is a complex process given that necrotic tumor cells produce various immune cell attractants and are, together with the leaked content, efficiently eliminated mainly by macrophages. This results in the digestion of the cellular DNA and the release of digested ctDNA into the extracellular space [15, 16]. For example, necrotic Jurkat T-lymphocyte cells (derived from a patient with T cell leukemia) alone released minimal levels of ctDNA, but coculture with macrophage cell lines caused a significant increase in ctDNA levels in the culture medium [40]. Detection of long ctDNA fragments might result from exceeding the phagocytic capacity in digesting the released cell contents [5, 40].

#### Other passive release mechanisms

##### Circulating tumor cells are a minor source of circulating tumor nucleic acids

CTCs are possible sources of ctDNA [15, 41, 42]. When CTCs are released into circulation, they face various biophysical pressures such as hemodynamic forces, bloodstream swiftness, collisions with blood elements, and complex formation with non-malignant cells such as leucocytes and thrombocytes [43, 44]. These can result in CTC breakage and release of NAs. But given the rarity of CTCs, the lack of evidence, and the infeasibility of quantifying the rate of these events, this proposed mechanism of ctDNA release may not be of clinical relevance [45]. Indeed, the ctDNA genome equivalents are 100 to 1000 times higher than those of CTCs, so the quantity of ctDNA does not correspond with the number of CTCs [4, 46, 47]. Also, ctDNA has often been present in samples where CTCs were undetectable but not vice versa [16].

##### Chromosomal instability can result in tumor DNA release from cells

Chromosomal instability represents a common trait of cancer and can result in the passive release of ctDNA [48–50]. CtDNA can be released via micronuclei, nuclear sub-entities containing chromosomal DNA that segregated aberrantly during mitosis and assembled their own nuclear envelope. Several micronuclei may be formed, with their levels increased in cancer cells. These sub-organelles have been hypothesized as possible translocators of DNA to the extracellular space [11]. Direct evidence studying isolated micronuclei is needed to confirm this potentially promising source of ctDNA [11].

Chromosome fragments that are not reintegrated into reassembled chromosomes can join together, creating double minutes (DMs). These tiny fragments of extrachromosomal DNA are frequently seen in many cancer types [51]. DMs have been observed as extrachromosomal circular DNA in mice and humans, often containing amplified oncogenes [11, 52]. DMs often lack regulatory sequences and are prone to continuous expression and autonomous replication leading to gene amplification. DMs can exit the nucleus by budding and subsequently be extruded from the cells as micronuclei [50]**.** Alternatively, micronuclei can be eliminated by autophagy [53] and DNA digested in lysosomes, eventually releasing ctDNA into the extracellular space.

##### The possible contribution of other cell death types to circulating tumor DNA release is unclear

Cell death is a complex process influenced by many factors and may be accomplished via different mechanisms [54]. Thus, the contribution of different cell death types to the ctDNA pool is difficult to estimate. An outstanding question has arisen, whether cell death types like parthanatos, pyroptosis, ferroptosis, necroptosis, and oncosis contribute to ctDNA release [5, 55]. Briefly, necroptosis is a caspase-independent type of programmed cell death possessing similarity to apoptosis but resulting in membrane rupture and cell content release. Similarly, ferroptosis is a membrane rupture-associated programmed cell death induced by the accumulation of lipid peroxides resulting from the failure of antioxidant glutathione-based systems. Pyroptosis is a caspase-dependent, rapid cell rupture-related form of cell death. Ischemic cell death, or oncosis, is a term for a lethal injury early response induced by ischemia [56] degradation by endonucleases and depends on the expression of specific proteins [57]. The role of these cell death types in ctDNA release is unknown and has yet to be demonstrated.

The rates at which different cell death types occur and contribute to the shedding of ctDNA are difficult to estimate. While specific cell death mechanisms are associated with distinct morphological, biochemical, and immune-related changes, these processes are molecularly interconnected [58, 59]. Crosstalk between cell death pathways occurs, allowing backup mechanisms to exist [60]. For example, the rate of necroptosis may be elevated in some cancers as an alternative cell death mechanism to apoptosis [61, 62], but it can also be attenuated in cancer cells resistant to cell death [61]. Cell death can be activated under specific conditions, e.g., stress [62]. For example, nutrient depletion often triggers increased rates of ferroptosis [59]. Parthanatos can be induced by DNA damage [63], and its rate is substantially elevated due to oxidative stress in the tumor microenvironment or by cancer treatment using alkylating agents [64]. Oncosis can frequently occur in cancers with the altered expression of ion channels and compromised ion gradient [65]. Pyroptosis, a type of cell death associated with inflammation, can be induced by damage-associated molecular patterns (DAMPs, e.g., cfNAs and other products of cell death) and can be observed more frequently in highly inflammatory cancers [66].

#### Passively released circulating tumor RNA is difficult to detect and analyze

RNAs are also released from the cells during cell death regardless of their type [67, 68]. Apoptotic bodies shed by Jurkat and HL-60 (promyelocytic cell line derived from human leukemia) cancer cells have been shown to carry rRNA, miRNA, and mRNA [68]. Thus, apoptosis has been proposed as a possible source of ctRNAs and apoptotic bodies as their protective carriers [31, 69]. This assumption was based on the observation of tumor-derived RNAs remaining stable in serum when associated with apoptotic bodies [70]. Also, passively released elements of cancer-specific small ncRNAs (termed orphan-ncRNAs, oncRNAs) have been observed [71].

Importantly, apoptotic bodies contain more likely randomly loaded residual RNA fragments [72] that are difficult to detect and analyze [69]. Moreover, apoptotic bodies, as well as necrosis-derived RNAs, are susceptible to fast digestion either during phagocytosis [15, 16, 31, 72] or by circulating ribonucleases [5, 27], and current data do not indicate if there is a clinically relevant portion of detectable cfRNAs originating from necrosis. Notably, free mRNA extracted from cells of human hepatocellular carcinoma cell line Hep G2 and added to healthy blood samples has been shown to be undetectable by subsequent polymerase chain reaction (PCR), suggesting RNA degradation by circulating ribonucleases [73].

### Active release mechanisms of circulating tumor nucleic acids and their properties

Studies have shown that active release can also be a significant source of ctNAs [25, 74, 75]. A study found apoptosis and necrosis rates do not correlate with cfDNA release from cultured cancer and non-cancer cells [76]. The same study also showed that cfDNA concentrations correlated with the percentage of cells in the G1 cell cycle phase and that cells in the G1 phase might shed cfDNA preferentially via exosome secretion, highlighting the importance of the active release [76]. Moreover, the presence of ctDNA in cell culture supernatant without cell death detection has been observed [75].

The active release is characterized by a homeostatic, regulated, and energy-dependent release of newly synthesized NAs. The process exploits proteins that execute the release of NAs from viable cells [17, 77–80]. A more accurate definition of active release mechanisms requires understanding the composition of secreted NAs, which is, together with the biological significance of actively released ctNAs, not well established [5, 17, 78]. The active secretion of ctNAs occurs via EVs, discussed in detail below, and protein complexes, which can potentially contribute to tumor invasiveness, progression, and therapy resistance [11, 25, 74, 76, 78]. EVs [17, 45, 70] and lipoprotein complexes [4, 74] are considered essential sources of ctRNAs protecting the cargo against degradation. Thus, we assume that active secretion is an important release mechanism for ctNAs, especially for ctRNAs, which are rapidly degraded when passively released.

#### Extracellular vesicles contain selectively secreted circulating tumor nucleic acids

EVs are a heterogenous population of mostly spherical lipid-bound particles acting as mediators of many physiological and pathological processes [4, 81]. Their release is believed to be beneficial for maintaining cell homeostasis and intercellular communication [82]. They contain NAs, proteins, soluble factors, receptors, and lipids depending on physiological conditions and reflect the composition of the cells from which they have arisen [77, 83]. EVs are coated with a lipid bilayer membrane which helps ctNAs to avoid degradation by nucleases and immune cells [84]. The assembly, release, and sorting contents of EVs are regulated by the cells of origin [77, 80]. Given that specific mRNAs and miRNAs are enriched in EVs, selective sorting of the EV’s cargo has been suggested [83]. Similarly, different EVs carry different regions of genomic DNA of the cell of origin [81, 85].

Tumor-derived EVs are known to promote tumor invasion, metastasis, and drug resistance since they can transfer tumor traits by entering other cells [86]. They also play a role in facilitating tumor cell migration [4, 85]. Thus, EVs are promising biomarkers with the potential to provide information about the tumor and its evolution.

The correlation between higher concentrations of tumor-derived EVs and increased tumor invasiveness has been reported in vitro and in vivo [4, 81, 87, 88]. Although the exact contribution of different types of EV-related NAs to ctNA release is not clear, double-stranded DNA (dsDNA) [89, 90], as well as single-stranded DNA (ssDNA) [91], have been shown to be associated with EVs. Additionally, cfDNA can be either attached to the surface of EVs [92] or embedded in their lumen [4, 5, 11]. Various ncRNAs and mRNAs have been shown to be present in EVs derived from tumor cells [90, 93, 94].

The International Society for Extracellular Vesicles (ISEV), a scientific organization that guides the research on EVs to advance the understanding of their biology, issued a guideline called Minimal Information for Studies of Extracellular Vesicles 2018 (MISEV2018) [95]. The guideline provides protocols and experimental approaches to elucidate EV-associated functional activities. The document also summarizes important aspects of EV research, including nomenclature, enrichment, and molecular characterization techniques [95]. Currently, three basic categories of EVs have been commonly distinguished: exosomes, microvesicles (MVs), and apoptotic bodies. All are known to be secreted abundantly in cancer patients and contain tumor DNA and RNA protected from extracellular digestion [4, 11, 81, 87]. Exosomes and MVs have been shown to be actively secreted (see discussion below), while apoptotic bodies released by dying cells are passively secreted.

##### Circulating tumor DNA associated with exosomes

Exosomes are the most studied entities among EVs [80, 96]. Their size varies between 30 to 150 nm, and their isolation is challenging due to the heterogeneity in size and antigen availability [77, 97]. They are formed endosomally in multivesicular bodies (MVB) and released via the fusion of the MVB with the cell plasma membrane. The formation of MVB is associated with extracellular messenger trafficking and maintenance of cell homeostasis [82]. Tumor cell-derived exosomes have been shown to promote tumor growth and progression, and their levels increased in the circulation of cancer patients [88]. Exosomes carrying cancer-derived molecular cargo have also been termed oncosomes [98]. Exosomal content reflects the cell of origin and its physiological state as cells regulate the incorporation of biomolecules into exosomes [77, 80]. Since the release of exosomes from both normal mammary epithelial cells and breast cancer cells inhibited further exosome release [88], tissue-specific feedback regulation of exosome release has been suggested [88].

**Fig. 2:**
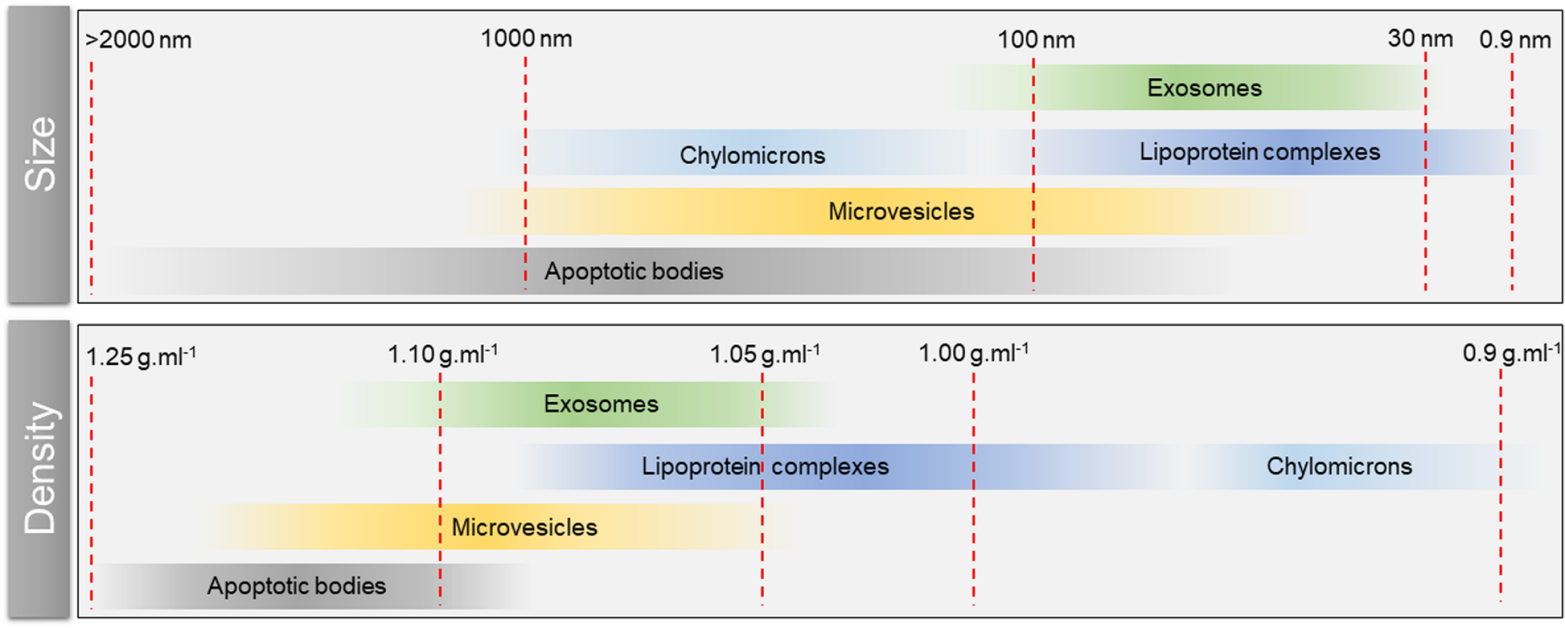
Heterogeneity of extracellular vesicles and lipoprotein complexes. Overlapping sizes and density hinder the separation and selective isolation of these components, and thus, their composition has yet to be fully described. A combination of multiple isolation techniques provides promising approaches for the comprehensive characterization of extracellular vesicles and lipoprotein complexes [99–101]

Notably, exosomal ctDNA has been observed to represent the whole genome and suggested as a clinically promising biomarker reflecting the mutation status of parental tumor cells [83, 89, 90, 92]. Interestingly, the majority of cfDNA (> 93%) has been observed to be localized within plasma exosomes [80]. However, a combination of high-resolution density gradient fractionation and direct immunoaffinity capture—a method that prevents aggregation of EVs during ultracentrifugation—showed exosomes not to carry dsDNA nor DNA-binding histones [99]. Thakur et al. [90] dispute these findings since they have observed tumor-derived exosomes carrying dsDNA. These contrasting findings indicate an urgent need to develop and standardize EV isolation techniques [83, 100, 101]. Different EV sizes (Fig. 2) might be related to varying amounts of DNA inside exosomes. Large EVs have been shown to contain a higher amount of tumor-derived DNA than smaller EVs [102]. Interestingly, smaller EVs have been observed to be more abundant than large EVs [102].

##### Microvesicles are a less studied but appreciable source of circulating tumor DNA

About 100 to 1000 nm in size, MVs are formed by the outward budding of the cell membrane [83]. MVs are also thought to contribute to cell homeostasis and cell-to-cell communication [94]. Less is known about their role in cancer, but MVs derived from cancer cells have also been shown to contain DNA [81, 83, 94, 103]. MVs contribute to tumor pathophysiology as mediators among cancer and stromal cell in the tumor microenvironment [85]. Moreover, the release rate of MVs has been shown to correlate with the progression of several cancer types [103].

Tumor-derived MV formation and release have been suggested to be associated with membrane regions rich in cholesterol and phosphatidylserine, which can act as promoters of shape changes of the membrane and detachment from the cytoskeleton [83]. ADP-ribosylation factor 6 (ARF6) has been demonstrated to be an important protein involved in the formation and shedding of MVs and the selective integration of their cargo. Moreover, ARF6 expression was associated with tumor invasiveness and detected on MVs released from a set of tumor cell lines [85, 94, 104]. Given that large MVs contain high amounts of tumor-derived DNA [102], we speculate that MVs might be a significant source of ctDNA. On the other hand, the broad range of size and density of MVs complicates their analysis (Fig. 2). Thus, the heterogeneity of MVs and low specificity of isolation techniques might compromise profiling experiments by co-isolation and analysis of different types of EVs carrying heterogenous cargos [83].

##### Circulating tumor RNAs are actively secreted via extracellular vesicles

EVs are considered an important source of ctRNAs [17, 45, 70]. Various ncRNAs and mRNAs are present in tumor-derived EVs [25, 84, 90, 93, 94, 105], including oncRNAs, breast cancer-specific ncRNAs thought to be involved in metastatic progression [71].

Increased levels of specific ncRNAs in exosomes in different cell states indicate a regulated sorting mechanism, possibly via sequences interacting with RNA-binding proteins and secondary/tertiary structures [96, 106]. The RNA-binding proteins, such as AGO2 [106], YB-1 [96], and nuclear ribonucleoprotein A2B1 [107], can mediate the sorting process and guide RNA to exosomes [96]. Membrane proteins, such as VPS4A and NSMASE2, have also been associated with higher levels of particular miRNAs in EVs [108]. Moreover, miRNAs can regulate the loading of other RNAs into the exosomes. For example, overexpression of post-transcriptional regulator miRNA-1289 increased the levels of *GALR3* G protein-coupled receptor mRNA directed into exosomes [109]. Specific CTGCC motifs and miRNA-1289 sequences have also been observed in many mRNAs enriched in EVs, including exosomes [110, 111]. Growth factors and nutrient deprivation have also been suggested as a stimulator of exosome secretion via the mTORC1 signaling [82]. Moreover, tissue-specific gene expression regulators, circular RNAs, have also been found and are stable in tumor-derived exosomes [86, 112].

Increased levels of specific ncRNAs in exosomes in different cell states indicate a regulated sorting mechanism, possibly via sequences interacting with RNA-binding proteins and secondary/tertiary structures [96, 106]. The RNA-binding proteins, such as AGO2 [106], YB-1 [96], and nuclear ribonucleoprotein A2B1 [107], can mediate the sorting process and guide RNA to exosomes [96]. Membrane proteins, such as VPS4A and NSMASE2, have also been associated with higher levels of particular miRNAs in EVs [108]. Moreover, miRNAs can regulate the loading of other RNAs into the exosomes. For example, overexpression of post-transcriptional regulator miRNA-1289 increased the levels of *GALR3* G protein-coupled receptor mRNA directed into exosomes [109]. Specific CTGCC motifs and miRNA-1289 sequences have also been observed in many mRNAs enriched in EVs, including exosomes [110, 111]. Growth factors and nutrient deprivation have also been suggested as a stimulator of exosome secretion via the mTORC1 signaling [82]. Moreover, tissue-specific gene expression regulators, circular RNAs, have also been found and are stable in tumor-derived exosomes [86, 112].

MVs derived from cancer cells have been shown to contain various tumor-derived RNAs [81, 103]. However, contamination by RNA from cell-free ribonucleoprotein complexes and exosomes cannot be excluded (Fig. 2) since protocols for selective isolation of EVs (MVs vs. exosomes) have yet to be standardized [113, 114]. Thus, although analysis of actively released ctRNA via EVs has potential as a cancer biomarker, there are technical challenges in the selective isolation of EV populations and their RNA content [115].

#### Actively released macromolecular complexes contain circulating tumor RNA

CtRNAs are also actively released as part of protein complexes [4, 11, 116, 117]. The population of circulating miRNAs independent of EVs has been observed in the human plasma digested by proteinase K [117]. Using non-cancer and cancer cell lines, miRNAs were shown to be associated with RNA-binding proteins protecting them from degradation [78, 117], such as AGO2 [117], but also other AGO proteins [116] as well as NUCLEOPHOSMIN 1 [78].

High-density lipoproteins (HDL) are other biomolecules that can form complexes with miRNAs and protect them against degradation [70]. The HDL-miRNA complexes have been suggested as a potential diagnostic marker in different pathological conditions, but the active and selective release mechanisms require more study [118].

## Factors influencing the release of circulating tumor nucleic acids

### Radiation therapy causes circulating tumor DNA release in a cell-type-specific manner

Radiation therapy induces necrosis and is considered a potential cause of necrotic cfDNA release. A transient rise in cfDNA levels has been observed after treatment, followed by a decrease after one or 2 weeks of treatment [13]. Interestingly, a 90% decrease in ctDNA levels has been shown after radiation therapy instead of an anticipated increase following cell death induction [119].

The decrease in ctDNA levels following radiation therapy has been proposed as an argument against necrosis as the main ctDNA release mechanism [74, 76]. However, the cell death mechanism depends on the cell type and molecular aberrations present in cells [120]. Radiation (or chemo) therapy might induce cellular senescence in one cell type and mitotic catastrophe in others [120]. These might be followed by late secondary apoptosis and necrosis [120, 121]. Alternatively, these therapies might cause an early release of cfDNA because of the initial high rate of apoptosis.

### Senescence as counteractor of circulating tumor DNA release

Cellular senescence is a permanent cell cycle arrest triggered by various intrinsic and extrinsic stimuli, such as DNA stress and damage during cytotoxic therapy [122]. Senescence has been observed as a potential counteractor of cfDNA release. Senescence induced by ionizing radiation resulted in a decrease in cfDNA release, while the induction of apoptosis in senescent cancer cells caused the opposite effect [120].

### Hypoxia as circulating tumor DNA release modulator in hypoxic tumors

Hypoxia, a state of reduced oxygen levels compared with its demand in tissues, is a canonical trait of cancer accompanying uncontrolled tumor growth. CtDNA levels in the blood of mice engrafted with TC1 epithelial lung cancer cells significantly increased after exposure to intermittent hypoxia [123]. Long-term hypoxia has also been shown to negatively modulate ctDNA release [124]. Thus, the rate of hypoxia, especially in highly hypoxic tumors, might have informative value for determining which tumors shed high amounts of cfDNA [4].

### Cell death can indirectly induce the active release of circulating tumor nucleic acids

Cell death is known for its association with the passive release of ctNAs [6, 8, 15, 72], but it can also cause an indirect induction of active release via paracrine signaling [55, 125]. Apoptosis has a pivotal role in carcinogenesis when not regulated and functioning correctly. But even if the apoptosis rate is normal or intentionally induced as part of anti-cancer therapy, it can cause the opposite of the desired effect resulting in apoptosis-induced proliferation (AIP). To keep tissues in their original state, a compensatory-regenerative program is triggered by the caspase-dependent release of mitogenic signals by apoptotic cells [125, 126]. EVs derived from apoptotic cells contain miRNA, long ncRNA, and mRNA, rendering them proliferative inducers and can turn into a vicious cycle when exploited by cancer cells [72, 127, 128]. A similar phenomenon can be found in necrosis induced by DAMPs. DAMPs include cfNAs and other cellular components released from necrotic cells capable of inducing tissue repair [30]. Both apoptosis and necrosis-induced proliferation may worsen inflammation and angiogenesis, contributing to more apoptosis and therapy resistance [30, 129, 130].

Thus, the relationship between apoptosis and increased ctNA levels might be more complex than commonly assumed. We speculate that part of this surge can result from increased cell proliferation induced by paracrine signaling and is associated with higher rates of active ctNA release. A question arises regarding how vital death-induced proliferation is in treatment resistance development. Apoptotic cells can stimulate adjacent treatment-resistant cells to grow [126]. However, AIP does not account for the presence of resistant cell-derived ctDNA [55]. Active secretion of ctDNA from these cells could partly explain this phenomenon.

### Molecular determinants of circulating tumor DNA release are poorly understood but are key factors in their release

Tumor molecular features associated with ctDNA release are underexplored areas of research [131]. Important associations have been recently reported. Transcriptional analysis of urothelial tumors from ctDNA-positive patients has shown higher cell-cycle and keratin gene expression levels, suggesting higher aggressiveness of the disease [132]. In the same study, tumors of ctDNA-positive patients without relapse have been shown to have increased expression of interferon-inducible genes. Interestingly, in lung cancer, tumor cell subclones carrying driver mutations have been shown to be more prone to release ctDNA when compared to subclones with nondriver mutations [131]. This study also demonstrated that subclones carrying mutations in cell cycle-related genes had relatively low ctDNA release efficiency. Thus, ctDNA release is strongly associated with tumor genetics and immunity, but molecular determinants of ctDNA shedding are poorly understood, and studies on other cancer types can provide novel associations.

## Implications of circulating tumor nucleic acid release on their properties and detection

**Fig. 3:**
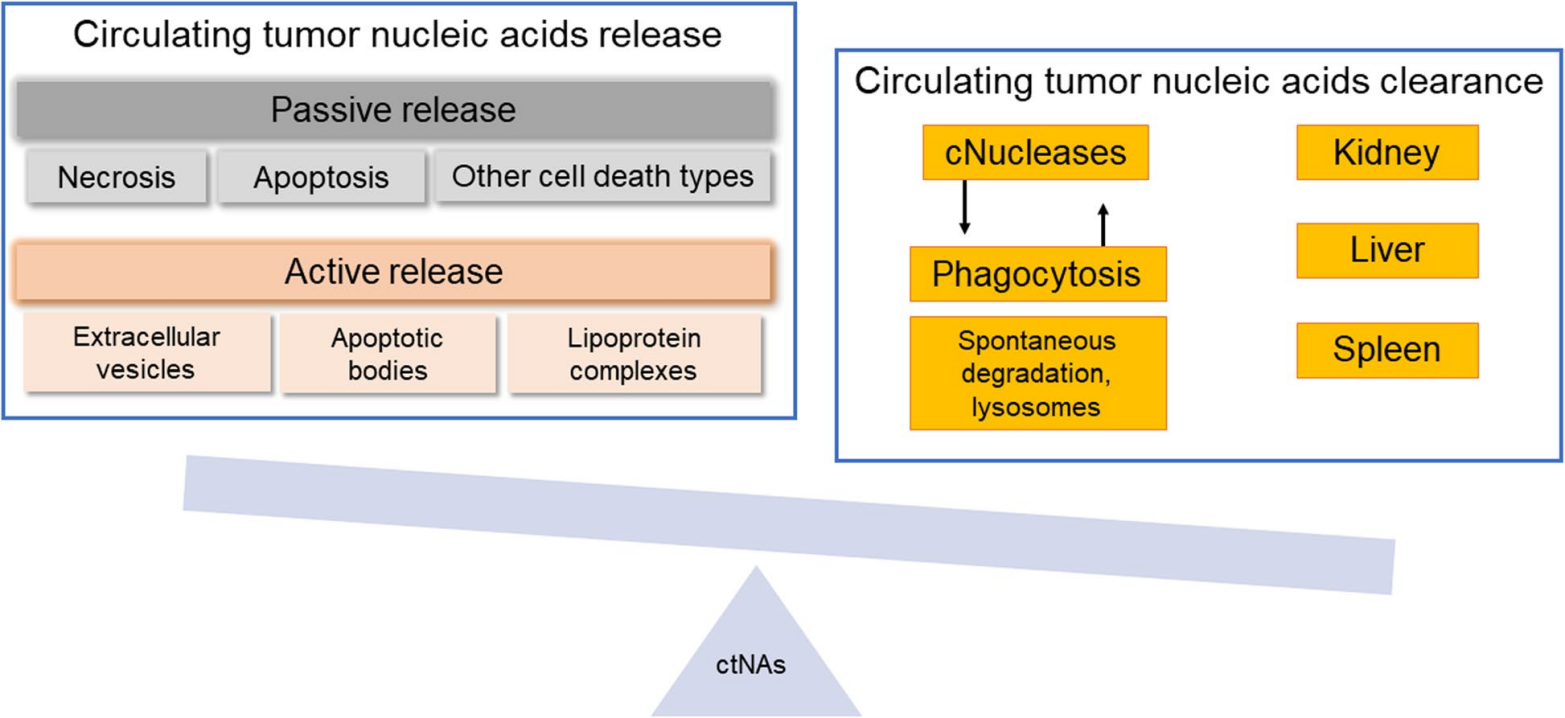
Factors determining the levels of circulating tumor nucleic acids. The presence of circulating tumor nucleic acids in the blood is determined by release mechanisms and their degradation and clearance (cNucleases – circulating nucleases)

To understand the clinicopathologic significance of ctNAs, it is essential to deeply understand ctNA release mechanisms (discussed above) and how the release and subsequent presence in the blood affect their properties. Thus, the understanding of ctNA degradation and clearance from circulation is pivotal to improved ctNA detection and analysis interpretation (Fig. 3) [15, 16, 120].

The estimated half-life of ctDNA in the circulation ranges from minutes to 1–2 hours [5, 16, 76, 133]. This fact points to ctDNA analysis as a real-time reflection of the disease. The duration of ctDNA half-life is associated with many factors, such as encapsulation in membrane-bound vesicles or association with protein complexes, tumor type, and treatment [5].

Interactions of DNA molecules with circulating protein complexes and serum proteins have been shown to affect the rate of clearance and degradation of cfDNA [4, 134]. Such macromolecular entities composed of cfDNA and other complexes (e.g., monoclonal antibodies, albumin, and nucleosomes) can impact the degradation of cfDNA by hampering the accessibility of DNases [135]. The clearance of cfDNA can also be affected if cfDNA is associated with EVs, as membrane encapsulation provides protection from degradation [134]. CfDNA uptake by different cells (adsorption to cell surface proteins and intake across the cell membrane) has also been suggested as a possible factor influencing the clearance of cfDNA [134, 136]. The clearance of cfDNA also depends on the physiological state of patients and associated factors such as surgery [136], sepsis [137], and tumor vascularization [134]. The factors modulating cfDNA levels need to be better understood [135, 136]. For example, the rate of DNA clearance through glomeruli has been shown to be dependent on the size of DNA molecules; however, more in vivo studies are needed to confirm this observation [5].

The vast size range of ctDNA molecules has been suggested to reflect enzymatic degradation and immune system clearance after both passive and active release [11, 16]. Additionally, together with the cfDNA pool, ctDNA is subjected to organ uptake and elimination mainly by the liver and the spleen and minimally by the kidneys [5, 11, 16, 45, 68].

### Circulating tumor DNA is generally shorter than cell-free DNA

While the ladder-like pattern in cfDNA size is widely accepted, ctDNA has been shown to be generally shorter than cfDNA. Increasing the PCR amplicon size from 82 to 181 bp in the detection of Epstein-Barr virus in the plasma of nasopharyngeal carcinoma caused an 87% decrease in its detection [138]. Also, analysis of DNA fragments shorter than 150 bp positively correlated with the tumor DNA fraction in the plasma [139]. In addition, a 2-fold median ctDNA enrichment (in > 95% of cases) was achieved by the analysis of cfDNA fragments in sizes from 90 to 150 bp [12, 35]. The improved copy number variant and single nucleotide variant detection method points to the advantage of analyzing shorter fragments in cfDNA. Thus, although previous experiments were focused on analyses of ctDNA fragments of about 150 bp, targeting shorter (< 145 bp) fragments might be vital to improving ctDNA detection and analysis [4, 33, 35].

The nature of ctDNA shortening needs to be better understood. It could be partly attributed to the DNA bound to transcriptional factors, which serve as additional protection against nuclease digestion, even for shorter sections of DNA (20–90 bp)[140]. Epigenetics might also be implicated in ctDNA shortening as a result of the tendency of hypomethylated regions to be less dense and less organized and, thus, more prone to nucleases digestion [12]. Additionally, tissue-specific processes, such as specific nucleosome wrapping, have been suggested as possible factors associated with different fragment length populations [141].

### RNA is subjected to intensive degradation early after apoptotic stimuli

There is strong evidence that cellular RNA is subjected to rapid degradation during apoptosis [142]. It has been shown that global mRNA decay occurs early after apoptotic stimuli before DNA degradation begins following the permeabilization of the outer mitochondrial membrane [142]. This permeabilization leads to the release of DIS3L2 and PNPT1 ribonucleases that degrade mRNA [142, 143], and thus, mRNA has been observed to comprise about 2.1% of the total extracellular RNAs [144]. Similarly, DIS3L2-associated degradation was also observed on pre-miRNAs and Poly(A) ncRNAs [145].

Unlike mRNA, short ncRNAs have been shown to be remarkably stable in the plasma and serum of cancer patients [70]**.** It should be noted that this is a peculiarity of shorter ncRNAs, such as miRNAs, while longer ncRNAs have comparable stability to that of mRNAs [146, 147]. The majority of ncRNAs are released as a part of EVs or protein complexes (as discussed in section "Actively released macromolecular complexes contain circulating tumor RNA"). These structures are rapidly cleared from the circulation [148] primarily by macrophages. However, the clearance also depends on complex immune pathways that stimulate phagocytosis [149]. Although liver and spleen macrophages have been observed to play a major role in the clearance of EVs from blood [149, 150], the mechanisms and factors influencing the clearance of ncRNAs from the blood have yet to be fully described.

## Circulating tumor nucleic acids as a reflection of tumor biology and evolution

### Circulating tumor DNA release as a reflection of tumor burden

Studies have revealed a positive correlation between tumor size and ctDNA quantity [47, 151]. Moreover, growing tumors, and thus an increase in the number of tumor cells, might produce more ctDNA, but there are also opposing data [4, 45, 47, 152]. For example, a study involving 640 patients with various cancer types (advanced pancreatic, ovarian, colorectal, bladder, gastroesophageal, breast, melanoma, hepatocellular, and head and neck cancer) revealed that ctDNA was detectable by digital PCR in > 75% of patients [47]. However, the rate of ctDNA detection was under 50% in primary brain, renal, prostate, and thyroid cancers [47].

The relationship between cancer burden and ctDNA levels is complex. Disease burden cannot be characterized only by the physical size of the tumor; this is due to the limitations of imaging techniques in accurately quantifying tumor size, especially in cases where tumors do not have distinct boundaries. Instead, characteristic tumor traits, like necrosis, have been shown to correlate better with ctDNA levels. A higher necrosis rate, typical for some cancers, is associated with higher stages of the disease and higher rates of ctDNA release [16, 153]. Indeed, lung squamous cell carcinoma with a higher necrotic rate has been observed to have higher ctDNA detection rates compared to adenocarcinomas [49]. Also, higher levels of ctDNA have been observed in triple-negative breast cancer (TNBC) compared to other subtypes [154]. The high rates of necrosis and cell proliferation in TNBC have been suggested as a rationale for these observations [18]. This might explain discrepancies in ctDNA levels across different cancer types and stages. Thus, understanding the biology and kinetics of ctDNA shedding in different cancer types might reflect tumor characteristics.

Cell doubling time, the number of proliferating cells, and tumor cell loss have been suggested as parameters affecting tumor growth kinetics [4]. Combining these parameters with ctDNA quantification might provide insights into the tumor evolution of specific cancer types. Similarly, a mathematical model for predicting detectable tumor size that considers tumor evolution and ctDNA release has been developed [155]. Using this model, the estimated % tumor cell genome released to the extracellular space per cell death was 0.014%.

### Circulating tumor DNA and cell-free DNA as a linked reflection of tumor biology

An increase in total cfDNA levels in cancer patients cannot be explained only by the fraction of ctDNA released [16]. CtDNA content in blood might vary due to inter-individual heterogeneity. The proportion of ctDNA within the cfDNA varies, ranging from 0.003 to 95% [4]. CtDNA levels are low in early-stage tumors, constituting up to 1% of cfDNA [10]. In patients with a high tumor burden, ctDNA may exceed 10% of cfDNA [35] and could reach up to 40% in the advanced stages of the disease [10]. To better understand tumor heterogeneity and evolution, both, ctDNA and cfDNA should be examined [4]. In addition to the release of ctDNA from tumor cells, infiltrating non-tumor cells in the tumor mass interacting with tumor cells might also die and release high amounts of cfDNA during the early stages of the disease [16, 156]. Thus, high ctDNA and cfDNA levels have been shown to correlate with high mutation load and cfDNA fragmentation rate [157].

### Long fragments of circulating tumor DNA as an indicator of a high necrotic rate

Necrosis is a characteristic cell death occurring in solid tumors [158]. The rate of necrosis varies among different cancer types [158]. Thus, discrepancies across ctDNA levels might occur, even in patients with tumors of the same type and stage [159]. Since necrosis is generally a faster and more disorganized process than apoptosis, large ctDNA fragments of kbps in size can potentially be released when the phagocytic capacity is overwhelmed [36, 81]. In tumors with a high necrosis rate, longer fragments can provide a clue for identifying the origin of cfDNA [16, 153]. Thus, the heterogeneity in ctDNA size could be a consequence of two independent factors in cancer: immune system efficiency and rate of necrosis [5, 40].

### Circulating tumor RNA as a reflection of tumor evolution

Studies have shown that changes in the expression profiles of miRNAs are associated with the development of metastasis and play a crucial role in tumor evolution via cell signaling [67, 160]. Thus, circulating miRNAs may represent molecular changes associated with tumor development and progression [160, 161]. However, inter-individual variability in the levels of circulating miRNAs in both cancer patients and healthy individuals and the lack of consistency in the analytical methods and conflicting results have confounded further understanding of their role in tumor evolution [67, 162, 163]. Circulating long ncRNAs are another class of emerging biomarkers comprising a large group of transcripts with diverse biological functions [164]. These molecules are transcriptional and posttranscriptional regulators functioning via interactions with DNA, RNA, and/or proteins [165]. They are expressed in concert with genes implicated in cell cycle regulation, survival, and pluripotency [166]. For example, circulating long ncRNAs MALAT1 (metastasis-associated lung adenocarcinoma transcript 1, a scaffold protein implicated in gene and splicing regulation) along with H19 and HOXA-AS2 (implicated in miRNA regulation) have been associated with proliferation, progression of the cell cycle, and cell migration [14]. Both circulating miRNAs and long ncRNAs are known to be associated with tumorigenesis in several cancer types [167]. Together, these circulating molecules can reflect the clonal evolution in the tumor [14, 166–168].

EVs play an essential role in tumor growth and evolution [169]. Exosomes have been shown to promote cancer via the transport of specific miRNAs that upregulate oncogenic pathways or by horizontal transfer of mutated mRNA cargo to non-cancer cells inducing tumorigenic transformation [77]. MiRNAs shed via tumor-derived exosomes may reflect molecular changes underlying tumor evolution [105].

## Clinical utility of circulating tumor nucleic acids

### Clinical utility of circulating tumor DNA

CtDNA-based liquid biopsy can provide minimally invasive and real-time assessment of tumor heterogeneity and treatment response as it exploits ctDNA released from different tumor subclones that might not be examined by locally limited tissue biopsy [6]. Mutations found in ctDNA have been shown to be concordant (up to 90%) with matched solid tumors [6, 152, 170]. Discrepancies between ctDNA and solid tumor tissue analyses are observed mainly in patients with low levels of ctDNA [152]. Since ctDNA may reflect systemic disease and are more abundant than CTCs [171, 172], the analysis of ctDNA could serve as a better measure of tumor burden and heterogeneity with higher sensitivity and specificity than the analysis of solid tumors [6] and CTCs [171].

Studies comparing primary tumors, CTCs, and ctDNA could provide a more comprehensive panel of biomarkers for disease monitoring. For example, in lung cancer, a study showed higher mutation detection by ctDNA analysis than CTCs or tumor tissue alone [172]. Another study demonstrated the feasibility of tracking tumor evolution dynamics using ctDNA [153]. Combined ctDNA and CTC analysis improved the sensitivity of primary lung cancer detection [173]. Also, the correlation between ctDNA presence vs. primary tumor proliferation index, invasiveness, and necrosis in non-small cell lung cancer (NSCLC) has recently been demonstrated [174]. In 20–30% of lung cancer patients, tissue biopsy is not practical due to insufficient tumor tissue available and the serious health risks it poses for some individuals [175]. Additionally, ctDNA has been shown to predict patient relapse in several cancer types, including lung cancer [176]. For example, NSCLC patients with serially undetectable ctDNA or with > 50% decrease in ctDNA levels post-treatment had longer survival than patients with detectable ctDNA or those with a lower reduction of in ctDNA levels [177]. Because of the ease and the feasibility of serial testing, ctDNA has become a valuable alternative to tissue biopsy for monitoring disease progression and predicting patient outcomes [153, 174].

CtDNA analysis could be implemented in multiple clinical settings: namely, screening and early detection of MRD, tumor characterization, treatment efficacy, and relapse monitoring [3, 8]. However, ctDNA analysis in each of these clinical applications has to be performed independently while corresponding preanalytical and analytical variables specific for each setting [178].

**Fig. 4:**
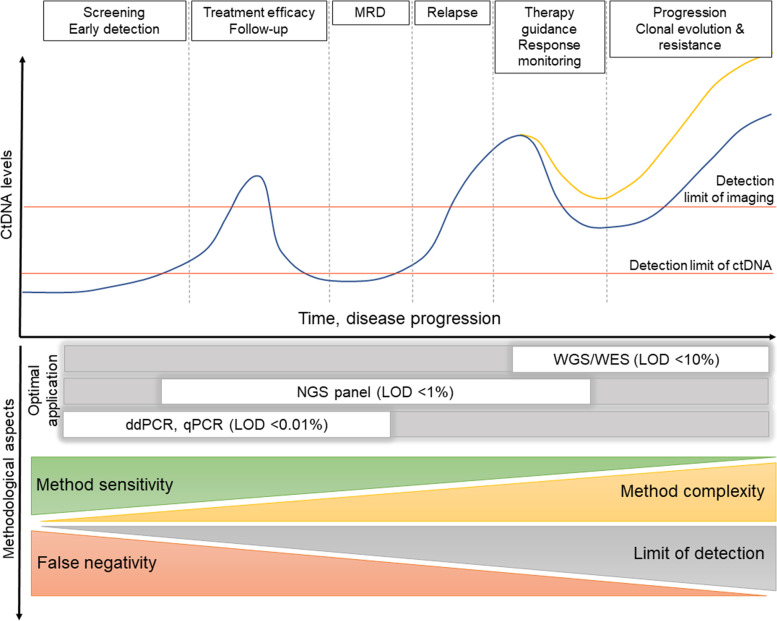
Clinical utility of ctDNA in the context of disease evolution over time. The blue line depicts the level of ctDNA that carries a mutation detected in both the primary tumor and the blood. The yellow line shows the level of ctDNA representing a mutation that emerged with treatment resistance. MRD – minimal residual disease, WGS – whole genome sequencing, WES – whole exome sequencing, NGS – next generation sequencing, LOD – limit of detection, ddPCR – digital droplet PCR, qPCR – quantitative PCR)

In addition to preanalytical variables that are known to affect the measurement of ctDNA levels (e.g., the choice of blood collection tubes, processing delays, sample volume, cfDNA isolation techniques, and quality control methods [179]), the impact of the timing of specimen collection on ctDNA analysis must be considered since ctDNA shedding fluctuates over time (Fig. 4) [4, 6, 169, 179–182].

CtDNA levels have been shown to correlate with tumor size, stage, and poor prognosis in many studies [4, 6, 47, 151, 154, 183, 184]. But data also show inter-individual variability among patients with the same cancer and across different cancer types rendering ctDNA evaluation challenging [4, 45, 47, 152]. In 2018, the American Society of Clinical Oncology (ASCO) and the College of American Pathologists (CAP) pointed to the lack of evidence of clinical validity and utility of the majority of ctDNA assays outside of a clinical trial due to discrepancies in their results [26]. Currently, ctDNA analysis possesses the highest clinical validity among liquid biopsy-based biomarkers and is closest to implementation in clinical practice. This is especially true for advanced disease genotyping, but its limitations must be considered in the data interpretation [178]. Several ctDNA-based diagnostic tests have been approved for clinical practice (Table 1), representing an important milestone in liquid biopsy implementation [6, 11].

**Table 1:** Approved diagnostic tests for detecting altered genes in cfDNA

Test	Approval	Cancer type	Analyte	Technology	Target
Cobas^®^ EGFR Mutation Test v2 [6, 185]	FDA, EMA	Non-small cell lung	Blood (cfDNA), tissue DNA (FFPE)	RT-PCR	*EGFR* gene
FoundationOne^®^ Liquid CDx [10]	FDA	Multiple	Blood (cfDNA)	NGS	Panel (300 genes)
Guardant360 CDx [186]	FDA	Multiple	Blood (cfDNA)	NGS	Panel (55 genes)
Qiagen *therascreen* PIK3CA RGQ PCR Kit [187]	FDA	Breast (selection of patients eligible for treatment with alpha-selective PIK3-inhibitor alpelisib)	Blood (cfDNA), tissue DNA (FFPE)	RT-PCR	*PIK3CA* gene
Epi proColon^®^ [188]	FDA	Colorectal	Blood (cfDNA)	RT-PCR	Methylated *SEPT9* gene

*FDA* Food and Drug Administration, *EMA* European Medicines Agency, *RT-PCR* real-time PCR, *NGS* Next generation sequencing

Tumor heterogeneity, evolution, and clonal hematopoiesis may partly cause ambiguity in the ctDNA measurement data [11, 189]. Still, the need for more standardization of both preanalytical phase and ctDNA detection methods remains the most significant challenge [189]. Interestingly, as the clotting process during serum preparation induces an increase in cell lysis, ctDNA analysis might be hampered by increased levels of high-molecular cfDNA when using serum instead of plasma [190]. Thus, plasma has been suggested as the better specimen type for the ctDNA analysis [5, 15, 26]. In addition, while EVs comprise a more consistent source of some miRNAs biomarkers, other miRNAs have been more efficiently isolated from plasma [191]. As described above, cfDNA occurs mainly as dsDNA [11, 89], and ctDNA is generally more fragmented than cfDNA. Since dsDNA library preparation might not detect highly degraded ssDNA, ssDNA-library-based sequencing might improve ctDNA recovery [12].

CtDNA analysis has the potential to provide valuable information regarding tumor dormancy. Actively released ctDNA may have clinical relevance in cancer patients at risk of having dormant disseminated tumor cells (DTCs) [120]. Given that senescent cells also produce EVs, mainly exosomes [192], these particles might be pivotal for dormant tumor cell detection [193]. Detecting dormant DTCs is technically challenging; thus, developing blood-based dormancy-related biomarkers (as surrogates of DTC presence) may improve sensitivities for the detection of MRD, which could be difficult to evaluate by conventional means, such as imaging.

Necrosis is associated with the release of large cfDNA fragments (up to kbps in size) from the cells [40, 86]. Necrosis-derived cfDNA is phagocyted and digested to residual fragments by macrophages [15, 16]. The presence of longer fragments of cfDNA in the circulation can indicate increased rates of tumor necrosis [4, 11]. Necrosis is related to the aggressiveness of the disease, and its increased rates have been associated with poor prognosis in several tumors, such as breast, renal, mesothelial, and lung cancers [158].

A ladder-like pattern of cfDNA sizes is a characteristic indication of apoptosis in cancer patients and healthy individuals [5]. As discussed previously, the cfDNA size profile has a size peak of 167 bp, corresponding to the length of DNA around one nucleosome with a linker DNA [33–35]. However, ctDNA fragments are shorter than cfDNA derived from non-cancer cells [32]. This is probably caused by cancer-related hypomethylation of DNA, which is more accessible to cleavage by nucleases [12]. Short cfDNA is more common in metastatic breast cancer patients when compared to primary breast cancer [32]. Moreover, specific size populations of cfDNA differ in genetic alteration frequency, and short cfDNA fragments have been identified as the major source of mutant-specific alleles [32]. The association of shorter DNA molecules to transcriptional factors [140] and tissue-specific nucleosome wrapping [141] can also explain ctDNA shortening (see section "Circulating tumor DNA is generally shorter than cell-free DNA"). Thus, the analysis of shorter ctDNA fragments (< 145 bp) may improve ctDNA detection among abundant cfDNA derived from non-cancer cells [4, 33, 35]. The different size profiles of cfDNA may serve as prognostic biomarkers as they vary in different stages and correlate with clinical outcomes [32]. Indeed, a correlation between shorter fragments of cfDNA and shorter progression-free survival and overall survival has been shown in pancreatic cancer patients [194]. Similarly, shorter cfDNA length was associated with poor survival and severity of renal cancer [195]. Altogether, the assessment of cfDNA levels and cfDNA size (e.g., shortening) correlates with advanced stages and cancer progression and thus could aid in predicting patient outcomes [32].

Additionally, tissue-specific DNA fragmentation and nucleosomal occupancy patterns have been proposed as promising tools for the identification of ctDNA tissue of origin [5, 16, 33]. Differentiation of ctDNA size populations might be of diagnostic value, which could improve ctDNA detection and cancer monitoring in different pathophysiological stages [4, 5, 141].

False positivity and negativity are critical challenges in the clinical implementation of ctDNA as a biomarker for guiding treatment and predicting recurrence [11, 15, 196]. False negativity may result from very low ctDNA content of cfDNA shed into circulation, especially in the early stage of the disease. Analysis of cfDNA fragment and epigenetic (e.g., methylation) patterns have been suggested as possible improvements to ctDNA analysis, providing reliable negative results [178]. Conversely, false positivity may arise from tumor heterogeneity but is more likely from clonal hematopoiesis and detection of somatic alterations in DNA released by normal blood cells [197, 198]. The predominance of cfDNA over ctDNA and its release mainly by hematopoietic cells, as well as the partial overlap of genes mutated in clonal hematopoiesis with tumor drivers, can significantly increase the risk of false-positive ctDNA detection and limit copy number alteration detection [178].

### Clinical utility of circulating tumor RNA

CtRNAs have been suggested as promising minimally invasive diagnostic and prognostic biomarkers [45, 70, 199]. For example, the levels of long ncRNA MALAT-1 (metastasis-associated lung adenocarcinoma transcript 1) detected in the blood of NSCLC patients reflected the presence of NSCLC with a specificity of 96% [200]. Higher levels of long ncRNA GIHCG (gradually increased during hepatocarcinogenesis) in the serum revealed renal cell carcinoma with a specificity and sensitivity of 84.8 and 80.7% [201]. Circulating long ncRNAs also have been suggested as potential prognostic biomarkers that can be used for patient stratification and prediction of survival outcomes [14, 202]. For example, increased expression of long ncRNA HOTAIR (HOX antisense intergenic RNA) in the blood of colorectal cancer patients positively correlated with higher mortality [203]. Also, high GIHCG levels correlated with poor survival in patients with hepatocellular carcinoma [204].

Aberrant expression of tissue-specific miRNAs has been suggested as candidates for cancer diagnosis, even for early-stage cancer screening [70, 205]. For example, significantly upregulated levels of serum miR-182, miR-183, miR-210, and miR-126 were shown to have diagnostic value for the early detection of NSCLC with a sensitivity of 81.3% and specificity of 100.0% when combined with carcinoembryonic antigen (CEA) [206]. MiRNAs miR-21-5p, miR-20a-5p, miR-141-3p, miR-145-5p, miR-155-5p, and miR-223-3p were significantly increased in the plasma of patients with stage I and II NSCLC [205]. MiRNAs also have prognostic value as they correspond to molecular changes and regulation of genes that promote disease progression [67, 105]. For example, increased plasma exosomal levels of miR-23b-3p, miR-10b-3p, and miR-21-5p were associated with poor overall survival in NSCLC [207]. Similar results have also been observed in other types of cancer (e.g., adenocarcinoma, myeloma, brain, colorectal, and breast cancer) [45, 70, 205]. In another study, a decrease in plasma levels of miRNA185-5p correlated with poor survival of patients with glioma [208]. In ovarian cancer patients, increased plasma miR-148a correlated with longer overall survival [205].

## Circulating tumor nucleic acids - current applications and considerations

CtDNA has been studied as a promising diagnostic, prognostic, and predictive biomarker for decades [4, 8, 15, 75]. More recently, research on ctDNA has accelerated following more extensive use of digital PCR and next-generation sequencing, thus improving our understanding of the origins of cfDNA and ctDNA [6, 10, 25]. The ctDNA population is heterogenous; it fluctuates among individuals with different but also the same cancer types and does not always correspond to the tumor burden [5, 47, 152]. CtDNA released because of cell death possesses a specific fragmentation pattern [11] and epigenetic signature [12]. While commonly observed to be associated with proteins, typically histones and transcription factors, ctDNA’s association with EVs requires further investigation [45, 99].

In contrast, ctRNA is distinguished by its association with EVs and lipoprotein complexes that serve as protection against degradation [45]. By using ctRNAs as a complementary biomarker, we can examine the expression signature of tumor cells and gain reflection of the tumor microenvironment and evolution, potentially filling in information gaps from ctDNA analysis alone. Hence, a multi-marker approach combining exosomal ctRNA and ctDNA might increase the sensitivity and relevancy of the analysis [6]. However, ctRNA has been studied less than ctDNA, and the isolation of RNA subpopulations derived from EVs and lipoprotein complexes remains a current technical challenge [17, 83, 114].

The properties of ctNAs, such as concentration, structure, and size, are determined in part by their release mechanisms and subsequent degradation and clearance from circulation [140]. Thus, it is crucial to understand these processes and how they affect the properties of released NAs [6, 8, 11, 17]. Several release mechanisms have been proposed for ctNAs, although their relative contribution to the resulting pool of ctNAs needs to be better understood [4]. CtNAs can be released via the passive mechanism associated with cell death, mainly apoptosis and necrosis. But passive release comprises just a portion of the total ctRNAs released [70], given that cellular mRNA can be subjected to early decay during apoptosis [142] and efficiently cleared by phagocytosis [16, 86]. In contrast, ctNAs can be shed into circulation by active release, an important source of stable forms of ctNAs, including ctRNAs [70, 96, 116, 117].

The abundance of cfDNA derived from blood cells can lead to decreased sensitivity in detecting ctDNA [83]. Moreover, preanalytical and analytical variability, together with biological heterogeneity (e.g., diverse and overlapping EV populations), can compromise detection experiments or prevent the comparison of data derived from different types of cancer [47, 83]. Thus, the standardization of preanalytical and analytical conditions for ctNA biomarker analysis is a crucial prerequisite for their clinical implementation [8, 15].

Despite persisting technical challenges, cancer heterogeneity, and the slow rate of new blood biomarkers approval, ctDNA analysis has been in the spotlight during the last decade [4, 6]. New protocols, independent parallel experiments, and regulatory guidelines have been suggested that consider validated pre-analytic and post-analytic principles of ctDNA analysis [8, 15, 26, 198]. Current proof-of-concept studies can lay the foundations for prospective studies with larger cohorts [6, 196, 198].

Moreover, international and interdisciplinary partnerships and consortia across academic institutions and industry have been established focusing on liquid biopsy implementation [8]— namely, SPIDIA4P consortium - Standardization and improvement of generic Preanalytical tools and procedures for In-vitro DIAgnostics [10], Cancer-ID [10], ISLB - International Society for Liquid Biopsy [209], ILSA - International Liquid Biopsy Standardization Alliance [7], ELBS – European Liquid Biopsy Society [8], BLOODPAC - US Blood Profiling Atlas of Cancer [8, 10]. The creation of these organizations is a crucial milestone for facilitating the standardization of ctDNA analysis for clinical applications.

## Future perspectives

We are witnessing an unprecedented development of liquid biopsy methods combining molecular biology, genetics, and computational approaches. This has resulted in the generation of vast amounts of data [6], enabling us to observe new associations between liquid biopsy-based biomarkers and clinical outcomes. In addition to somatic mutations, fragmentomic [12, 210] and epigenetic features [12, 33] of cfDNA have emerged as promising detection targets closely reflecting the tissue of origin. However, technical challenges impede their use in clinical practice. Although not yet fully implemented in clinical practice, machine learning algorithms are promising tools that might facilitate the clinical use of epigenetic and fragmentomic features of ctDNA [12, 211] as well as cancer-related ctRNA signatures [212]. The clinical implementation of ctNA data can lead to routine preventive screening for predisposition to cancer [178, 211, 212], monitoring drug efficacy, and predicting the potential for distant recurrence. CtDNA analysis is a promising tool with the potential to transform cancer diagnosis and management. However, the advantages and persisting limitations must be considered when applying ctDNA analysis in clinical settings. Future studies focusing on ctDNA and ctRNA release mechanisms might elucidate their role in tumor evolution and treatment resistance and overcome current limitations for clinical implementation.

## Conclusions

In conclusion, recent developments of advanced technologies with exquisite sensitivities have helped uncover the uniqueness of each tumor and the molecules they shed into circulation. The diagnostic, predictive, and prognostic value of blood-based biomarkers can be exploited for personalized medicine to improve cancer patient outcomes. In this review, we have described the mechanisms involved in ctNA release as well as the biological and clinical aspects of their detection. Since understanding the nature of ctNAs is a prerequisite for improved data interpretation, identification of treatment responsive versus resistant cell populations, and demonstration of the clinical utility of ctNAs, future studies focused on their biology are needed.

## Data Availability

Not applicable.

## Abbreviations

AIP: Apoptosis-induced proliferation
ARF6: ADP-ribosylation factor 6
ASCO: American Society of Clinical Oncology
CAD: Caspase-activated DNase
CAP: College of American Pathologists
CfDNA: Cell-free DNA
CfNAs: Cell-free nucleic acids
cNucleases: circulating nucleases
CTCs: Circulating tumor cells
CtDNA: Circulating tumor DNA
CtNAs: Circulating tumor nucleic acids
CtRNA: Circulating tumor RNA
DAMPs: Damage-associated molecular patterns
ddPCR: digital droplet PCR
DMs: Double minutes (joined chromosomal fragments)
dsDNA: double-stranded DNA
EMA: European Medicines Agency
EVs: Extracellular vesicles
FDA: Food and Drug Administration
HDL: High-density lipoproteins
LOD: Limit of detection
MRD: Minimal residual disease
mRNA: messenger RNA
MVB: Multivesicular bodies
MVs: Microvesicles
NAs: Nucleic acids
ncRNA: non-coding RNA
NGS: Next generation sequencing
oncRNAs: orphan non-coding RNAs
PCR: Polymerase chain reaction
qPCR: quantitative polymerase chain reaction
ssDNA: single-stranded DNA
WES: Whole exome sequencing
WGS: Whole genome sequencing
